# Assessment of Simple Gait Related Dual and Triple Tests in Predicting the Risk of Fall in Adults Above Age of 50 years

**DOI:** 10.7759/cureus.651

**Published:** 2016-06-22

**Authors:** Swati Paranjape, Disha Chitalia

**Affiliations:** 1 Physiotherapy Department, King Edward Memorial Hospital and Seth Gordhandas Sunderdas Medical College; 2 TNMC Nair Hospital Mumbai

**Keywords:** tug, dual task, triple task, risk of falls, elderly, timed up and go test

## Abstract

Timed UP and Go Test (TUG) is conventionally used as predictor of falls in adults. Routine daily activities include multiple tasks performed concurrently. When two or more tasks *(Dual/Triple test)* needed to be carried out concurrently, task performance declined at least in one of them. Our study aimed to find temporal and demographic variations in the performance after adding a cognitive, motor or both tasks, while performing TUG, compared to performance during conventional TUG. Sixty randomly selected healthy adults, with age ranging from 53 to 90 years, consented to participate in the study. Each participant underwent six tests (*Conventional TUG, Motor TUG, Cognitive TUG, Motor and Cognitive TUG, Visuospatial TUG, Motor and Visuospatial TUG*), with time measured in seconds. 6 (10%) had a previous history of fall. *Triple test *identified the highest number of participants at risk of fall (16.67%). ​One way ANOVA test showed significant temporal variation with the addition of task (p value< 0.0002). There was moderate positive correlation of age with the time taken to perform each test with addition of task. Conventional TUG in itself was found to be most sensitive and specific test to identify fallers. Though dual and triple task tests were also comparable, addition of task to TUG is not a sensitive indicator to identify fallers as compared to TUG.

## Introduction

Each year, one in three adults aged 65 and older, suffers a fall [1]. The number of falls keep rising with increased longevity of the population. Conventionally, the ‘Timed Up and Go’ test (TUG) is used as a predictor of fall in adults [2]. TUG is easy to perform and has a reported sensitivity of 80% and specificity of 94% [2]. Activities of daily living do not limit to only one task at a time. We usually perform *'dual tasks'*, like holding a cup of tea while walking or communicating with someone while walking; as well as *'triple or multiple tasks'*, like crossing an obstacle while holding an object and talking. Various studies have assessed the performance of dual and triple tasks and their influence on prediction of falls. Conventional TUG, motor TUG and cognitive TUG were comparable in determining the likelihood of falls [3]. Motor dual task TUG better identifies pre-frail individuals than single task TUG. Motor TUG was found to be more valid and sensitive than conventional TUG, therefore it can serve as screening tool for early detection of individuals with frailty in community dwelling adults [4]. The addition of dual task or cognitive task to TUG, improves the detection of risk of falls in people with Parkinson’s disease [5].

Thus, our study aimed to determine the temporal and demographic variations in the performance outcomes of dual and triple task TUG against conventional TUG, amongst community dwelling Indian adults above 50 years of age. Secondarily, the study involved classifying them as one of three categories, namely: a) at no risk of fall, b) high risk of fall, c) definite fallers, based on each test outcome measures. Thereby, we propose an alternate hypothesis i.e. demographic and temporal variations are seen while performing dual and triple tasks in community dwelling Indian adults and a null hypothesis i.e. there is no demographic or temporal variation seen while performing dual and triple tasks in community dwelling Indian adults.

## Materials and methods

This cross-sectional analytical study was done at the physiotherapy department of a tertiary care centre. Sixty healthy adult participants, with age greater than 50 years, presented to the physiotherapy outpatient department as accompanying relatives of patients coming for therapy, were randomly selected. Participants were excluded if they had any form of neurological deficit, which could lead to imbalance, in-coordination and dementia, like stroke, Parkinson’s disease, head injury, brain tumours and vertigo. Conditions for exclusions included any upper or lower extremity musculoskeletal deficit or congenital deformity; affected vision, even with the assistance of visual aids; inability to count. Institutional ethics committee approved the research protocol for the purpose of the study. Written informed consent was taken from all the participants of the study at the time of enrolment.

Detailed history and demographic findings were recorded for all participants at the time of enrolment in the study. The study protocol and the task procedures were explained to each participant. Each person was asked to perform six different tasks: 1) *conventional TUG*; 2) walking with a cup filled with water *(TUG motor)*; 3) counting backwards from 100 while walking *(TUG cognitive)*; 4) holding a cup filled with water and counting backwards while walking *(TUG motor and cognitive)*; 5) stepping over an obstacle while walking *(TUG visuospatial); *6) stepping over an obstacle while walking holding a cup filled with water *(TUG motor and visuospatial)*. The outcome was measured in terms of time taken to perform each task; calculated in *seconds (sec)*. A washout period of 20 minutes was given between each task. The tasks were performed in a well-lit and well-ventilated room with a three meter even pathway. An arm rest chair was used with a seat height of 43-46 cm and arm rest height of 62-66 cm [2]. An empty shoe box, 10 cm high, 19 cm wide and 33 cm long was used as an obstacle to cross [7]. A cup filled with water was used. A three meter pathway was marked. The chair with arm rest was kept at the starting line. For the conventional TUG, participants seated in the chair were asked to get up from the chair with the word ‘Go’, walk the pathway till the marked line, turn around, walk back and sit on the chair. Time was recorded with a stopwatch starting from the command word ‘Go’ till the participant completed the task and their buttock touched the chair again. After a wash out period of 20 minutes each, they were asked to perform above mentioned dual and triple tasks along with the conventional TUG procedure.

Cut-off values were set to identify the persons with risk of fall: 13.5 sec for conventional TUG [2,6]; 14.5 sec for TUG with any manual task (*dual test*); 15 sec for TUG with any cognitive task (*dual test*) [3]; 16 sec for TUG with any triple task (*triple test*) [3,8]. Cut-off value for triple test was calculated as an additional time of 22% per addition of each task [5]. Cut-off value for definite fallers (needing assistance) was set at 30 sec [2].

Statistical analysis was done using the Graphpad Prism 6 for Windows (publisher GraphPad Software), using one way ANOVA, with post hoc Newman-Keuls multiple comparison test. The sensitivity, specificity, positive predictive value and negative predictive value were calculated using standard formulae within the analysis software.

## Results

Sixty participants were included in the study, age ranging from 50 years to 93 years (mean- 65.5 years). There were 19 females and 41 males, in a ratio of 1:2.2. Four participants (6.67%) used a walking aid (a stick). Six participants (10%) had a previous history of fall, while 90% presented without any precious history of a fall.

Table 1 demonstrates the distribution of patients into the categories of no risk of fall, high risk of fall and definite faller, based on the results of the TUG tests. TUG motor *(Dual test)* and TUG with motor and cognitive *(Triple test)*, identified the maximum participants with high risk of fall. (15%, n- 9/60), while TUG with motor and visuospatial *(Triple test)*, identified the maximum number of participants who were definite fallers (3.33%, n- 2/60). TUG with motor and cognitive task *(Triple test)* identified the highest number of participants at risk of fall, 16.67% (n-10/60 = 9 with high risk of fall + 1 definite faller).

**Table 1:** Distribution of participants into categories of risk of fall as per the tests’ outcome

Tests (n=60)	No Risk of Fall	High Risk of Fall	Definite Faller
Tug	54	6	0
Tug motor	53	7	0
Tug cognitive	51	9	0
Tug visuospatial	52	7	1
Tug motor and cognitive	50	9	1
Tug motor and visuospatial	51	7	2

Table 2 shows the mean time taken to complete each test. Addition of motor and cognitive task to TUG, in *Dual and Triple tests*, is shown to take increased amount of time to complete the test, with the minimum time taken for conventional TUG (mean- 10.39 secs) and maximum time taken for TUG with motor and cognitive (mean- 12.73 secs).

**Table 2:** Mean time taken to complete each test

Tests	Mean Time (Secs)
Tug	10.39
Tug motor	11.42
Tug cognitive	11.73
Tug visuospatial	11.41
Tug motor and cognitive	12.73
Tug motor and visuospatial	12.50

Spearman correlation coefficient between the age and the time taken to perform each test is summarized in the Table 3. The summary shows that there is moderate positive correlation between the age and time required to complete each test. With advancing age, time taken to perform the tests increases.

**Table 3:** Correlation of age with time taken to complete each test

Tests	Correlation Coefficient
Tug	0.66
Tug motor	0.70
Tug cognitive	0.65
Tug visuospatial	0.64
Tug motor and cognitive	0.66
Tug motor and visuospatial	0.75

Assessment of the temporal variation between each test, using the one way ANOVA test showed a significant temporal variation (p < 0.0002) between all the tests. Newman-Keuls multiple comparison test showed significant temporal variation between TUG and *Triple tests*. This supports our hypothesis that there is temporal and demographic variation seen with addition of dual or triple task to TUG.

The sensitivity, specificity, positive predictive value and negative predictive value of each test are summarized in Table 4. All tests were equally sensitive in predicting the risk of falls. However, conventional TUG was found to be the most specific test, with the *Dual *and* Triple tests* being almost equally comparable with conventional TUG. Conventional TUG had the best positive predictive value for the prediction of falls, while TUG with motor and cognitive, had the least positive predictive value. All tests were equal in their negative predictive value for the prediction of falls in healthy community dwelling adults above 50 years age.

**Table 4:** Sensitivity, specificity and predictive values of the tests

Tests	Sensitivity	Specificity	Positive Predictive Value	Negative Predictive Value
Tug	1	1	1	1
Tug motor	1	0.96	0.75	1
Tug cognitive	1	0.94	0.66	1
Tug visuospatial	1	0.98	0.85	1
Tug motor and cognitive	1	0.92	0.6	1
Tug motor and visuospatial	1	0.94	0.75	1

## Discussion

As age advances, it is difficult for the elderly to coordinate two streams of visual information i.e. one related to navigation through visually defined space and the other related to a visually demanding second task [6]. When two or more tasks are needed to be performed concurrently, the task performance declines in at least one of them. Neuronal plasticity may help overcome this problem in the older age group, but it is at the cost of cognitive resources. They are no longer available for other activities while walking, such as obstacle avoidance, navigation along planned route, watching for pedestrian and vehicular traffic, as well as engaging in tasks not related to gait. As a consequence, the elderly often face more difficulties than the younger ones, in walking and concurrently engaging in another activity [6].

The study showed that the age has moderate positive correlation with the time required to perform each test. Thus, an increase in the age, moderately increases the time needed to complete a particular task. However, increasing age is not the only factor responsible for the test performance and risk of falls. Falls can occur because of various intrinsic and extrinsic factors [8]. Intrinsic factors include balance impairment, neurological disorders, sensory deterioration, musculoskeletal disorders, postural hypotension and medications which are used by the person. Extrinsic factors include ill-fitting footwear, poor lighting, surface quality and inappropriate furniture. Fallers show more evidence of cognitive impairment, use of diuretics and tranquillizers [9]. Present study was performed on healthy individuals and in a controlled environment; thereby the influence of such intrinsic or extrinsic factors could not be accounted for. There was a significant temporal variation with the addition of tasks in all age groups i.e. there was significant increase in the time taken to perform the TUG with additional task. Balance deteriorates in elderly when sensory inputs contributing to balance control are reduced, indicating that balance depends not only on motor, but also sensory system functions. In recent years, however, it has become increasingly apparent that other neural systems, including cognitive resources, may contribute to balance control [10, 11]. Additional research has shown that, with a simultaneous walking and talking task, participants were found to either stop talking or take longer time to complete gait task [11]. This was evidenced on our study as the participants taking increased time to perform each additional task, thereby supporting our alternate hypothesis. These findings confirm the notion that the balance performance is influenced by simultaneously performing cognitive and motor tasks. Lundin-Olsson et al also found that those who are more distracted by a familiar manual task performed concurrently with functional manoeuvres, were more at risk of fall [12].

All tests were found to be comparable in their efficacy, with sensitivity and specificity above 90%. This supports the findings that all the tests are comparable. Measurement of mobility and balance under multitasking conditions is not a more sensitive indicator of likelihood for falls. Therefore adding a task was not found to have any added advantage in identifying high risk or definite fallers. The study highlights the potential importance of dual/ multi-task balance training as a preventive measure in elderly, as the findings of the study showed an increase in performance time with addition of tasks to conventional TUG.

### Limitations and future directions

The study included only healthy individuals and their performance in a controlled environment, so the influence of other intrinsic and extrinsic factors, on test performance, could not be assessed. A follow up on the assessment of the participants and their falls was not performed. There lies a potential for future research in examining the effects of preventive dual/ multitask balance training on the test performance.

## Conclusions

The time needed to perform the TUG, dual or triple tasks increases with advancing age. Factors other than age may also play an important role in maintaining balance and increasing the risk of fall. Conventional TUG is the most sensitive and specific test to identify fallers. Though dual and triple task tests were also comparable, addition of a task to TUG did not turn out to be a sensitive indicator to identify fallers as compared to TUG.
